# Topics of the Month

**Published:** 1895-01

**Authors:** 


### TOPICS OF THE MONTH.

Mr. Lawson Tait, whose critique on the germ theory of disease was published in the December issue of the Journal, requests us to say that the mortality of his perineal operations should be *four* instead of *two*, as stated on page 277. Two fatal cases were overlooked in the preparation of the paper, for which omission Mr. Tait desires to apologize to our readers.

---

Dr. A. Conan Doyle, the distinguished literateur, has recently spent two months in the United States, during which time he has visited several cities of importance. He seems to have been more impressed with Philadelphia than with any other American city.

On the evening before his departure for home he was tendered a farewell dinner by the Aldine Club of New York, which had also similarly entertained him on his arrival in this country.

Hamilton Mable, as president of the club, presided and introduced the guest of the evening, whose presence, he said, represented an international affair, because Dr. Doyle was an Irishman by blood, was born in Scotland and spoke English.

Dr. Doyle's remarks were inclined to be of a retrospective character, and, judged from them, his American retrospect is a sincerely pleasant one. He said he felt like a hurdler who had taken all the obstacles, and they were represented by the East, the West, the North and the South of this broad land, but the greensward between would always appear to him as the Aldine Club. It would require the quiet of his study to separate the powerful impressions he had received while in this country. At present they were only a chaotic mass. The panorama which was whirling in his mind contained visions of the Hudson, with its chain of old Dutch towns ; a delightful glimpse of the industry of the manufacturing West, and another of Chicago, which reminded him of a half-grown boy who was always outgrowing his clothes. A view of staid old Philadelphia brought back placid memories of rural England, and so he went on describing briefly all the cities he had visited, and ended by saying that when he left he should leave a part of his heart behind.

Sir Henry Cunningham, the English author, paid a loving tribute to the memory of James Russell Lowell, and other speeches were made by "Bill" Nye, F. Hopkinson Smith, David Christie Murray, the Rev. Dr. Henry Van Dyke, Thomas Nelson Page, Frank R. Lawrence, Ripley Hitchcock, Captain J. T. J. H. Doyle, John Burroughs, Noah Brooks, William Cary and others.

---

It is proposed that a testimonial, to take the form of a portrait, shall be presented to Sir Joseph Lister, as a manifestation of the esteem in which he is held by his former colleagues and pupils. His recent retirement from active hospital and teaching work has been thought an appropriate occasion for the presentation. Subscriptions have been limited to two guineas (about $10.23) and it is hoped that sufficient funds will be collected to permit the presentation of some memento of the occasion to each subscriber.

Subscriptions may be sent to J. Frederick W. Silk, honorary secretary, 29 Weymouth street, Portland place, London W., England, or to one or other of the following gentlemen, who have kindly consented to act as treasurers, namely—Dr. James Finlayson, 4 Woodside place, Glasgow, Scotland ; Professor Chiene, 26 Charlotte square, Edinburgh, Scotland ; Professor William Rose, 17 Harley street, London W., England ; Dr. Malloch, 124 James street south, Hamilton, Ont., or J. Stewart, M. B., 37 South street, Halifax, Nova Scotia.

---

The Charity Organization Society, of Buffalo, makes an earnest appeal to our citizens for funds to meet its present necessities. It needs a permanent addition of, at least, $3,000 to its annual subscription list. It also needs to raise $5,000 extra to pay off its accumulated indebtedness. The extraordinary demands made upon the resources of the society, owing to the financial stringency of the past year and a half, has made this appeal necessary.

We trust the good people of Buffalo will unite in helping to raise these funds at once. In another place in this issue of the Journal we have presented cogent reasons in addition to those usually given why this should be done. Subscriptions should be sent to Mr. Frederick Almy, secretary and treasurer.

---

The propriety of abolishing the evening office hours of physicians is being agitated by some of our contemporaries. We quite agree with that spirit which would release the doctor from the thralldom of rigidly keeping evening hours at his office, when he could employ the time to better advantage in study or in a social manner.

In the few instances where it is impossible for patients to attend in the day-time, special evening appointments may be made ; but we are quite of the opinion that the abolition of evening office hours in general would result in improvement to both physicians and patients.

---

Georgia is struggling over the question of a medical practice act, which, it is to be hoped, will become a law during the present session of the state legislature. The speaker's vote was necessary to carry it in the house, but according to the *Atlanta Medical and Surgical Journal* it is believed that there will be very little opposition to it in the senate. The time is not far off when every state in the Union will enjoy the protection which a separate medical examining and licensing board affords.

The New York Medical College, which began its existence in 1850 and was discontinued in 1864, boasted of many distinguished alumni. In the list of graduates lately published may be found the names of Alexander B. Mott, Fessenden N. Otis, John Byrne, Edward S. Dunster and Alexander Hutchins.

A sketch of this college was prepared by the late Edwin Hamilton Davis, M. D., and has been published by his widow for the purpose of making the history of the college complete and publishing to the world its legally graduated alumni.

Every alumnus of this institution will be interested in reading this well-prepared little brochure.

---

The attention of our readers is invited to the excellent report of the eighth international ophthalmological congress held in Edinburgh, August 7-11, 1894, prepared by Dr. A. A. Hubbell, of Buffalo. The publication of the proceedings was commenced in the December number, is carried on in this issue and will be continued in succeeding months until completed.

The editor of the *Ophthalmic Record* (Nashville, Tenn.,) announces in a late issue that it has secured the services of Dr. Hubbell to report the proceedings of this congress for its pages; whereas, as a matter of fact, Dr. Hubbell's report is made exclusively for the Journal, and by permission will be printed in the *Record.*

---

Dr. Ernest Wende, Health Commissioner of Buffalo, has asked the Common Council to appropriate $2,700 to enable him to employ two bacteriologists, whose duty it shall be to aid in the manufacture of antitoxin and to investigate infectious diseases referred to them through the Health Department.

This reasonable request should be granted. Deaths are constantly occurring from diphtheria and other communicable diseases that are preventable. New York City has appropriated $75,000 to enable its health department to manufacture antitoxin, which is more than double the sum that the entire health department in Buffalo costs each year.

There is no excuse for delay in this matter. Infectious communicable diseases that are preventable should be restrained by every known method. Every element whose usefulness has been demonstrated by modern scientific research should be employed to prevent the spread of such diseases as diphtheria, consumption, typhoid fever and the like. Buffalo with upwards of 300,000 inhabitants cannot afford to take less than the most advanced stand in this matter.

Dr. Wende, as head of the Health Department, is asking for very little and he should get it promptly.

---

The treatment of insomnia is an interesting subject to every physician, and any methods looking to its relief always attract attention. The Medico-Chirurgical Faculty of Maryland lately had the subject under discussion, in which Dr. E. N. Brush, formerly of Buffalo, but now superintendent of the Sheppard Hospital for the Insane, Towson, Md., took an active part. He called attention to the evils that arise from the constant administration of drugs, especially where they are resorted to independently of the advice of a physician.

Dr. Brush is a strong advocate of natural methods to invoke sleep in cases of physical and nervous exhaustion, such as attention to the skin and other emunctories, massage, baths and the ingestion of proper food.

This is most excellent advice and is especially timely during a period when so many hypnotic drugs are being manufactured and sold with so much recklessness as to dismay almost every regular practitioner of medicine.

---

The report of the Buffalo Free Kindergarten Association, May, 1893, to September, 1894, has been issued. It makes a creditable showing of excellent work done in spite of the limited means at command. There are enrolled over 600 children, between the ages of three and seven, in the ten kindergartens of the association.

In this large city the association has less than two hundred members, though the annual dues are only $5.00. An increased membership is necessary in order to secure the continuance and extension of the work. This is one way to prevent sickness and reduce the death-rate, for everything which contributes to cleanliness and mental improvement serves to prevent squalor and disease.

No more tender appeal could be made to humanity than this one on behalf of the poor children of the city. Those who desire to become members may send their fee to Mr. Worthington C. Miner, treasurer, or to Mr. John G. Milburn, president.

---

The Postmaster-General, in his last annual report, pays particular attention to the abuses connected with second-class mail matter. These abuses consist chiefly of two kinds—namely, one where books, falsely purporting to be periodicals such as serial paper-covered books, are posted in this class by great publishing houses; the other in the mailing of sample copies.

There is no doubt that this latter evil is a serious one, and we wish the privilege of mailing sample copies at pound rates would be denied. The present practice, as the Postmaster-General says, offers a premium to worthless advertising sheets, and if it were discontinued the field for journalistic enterprise would be left open to legitimate publications, which, depending on subscriptions as well as on advertising, would go to those only who cared to take them on their merits.

